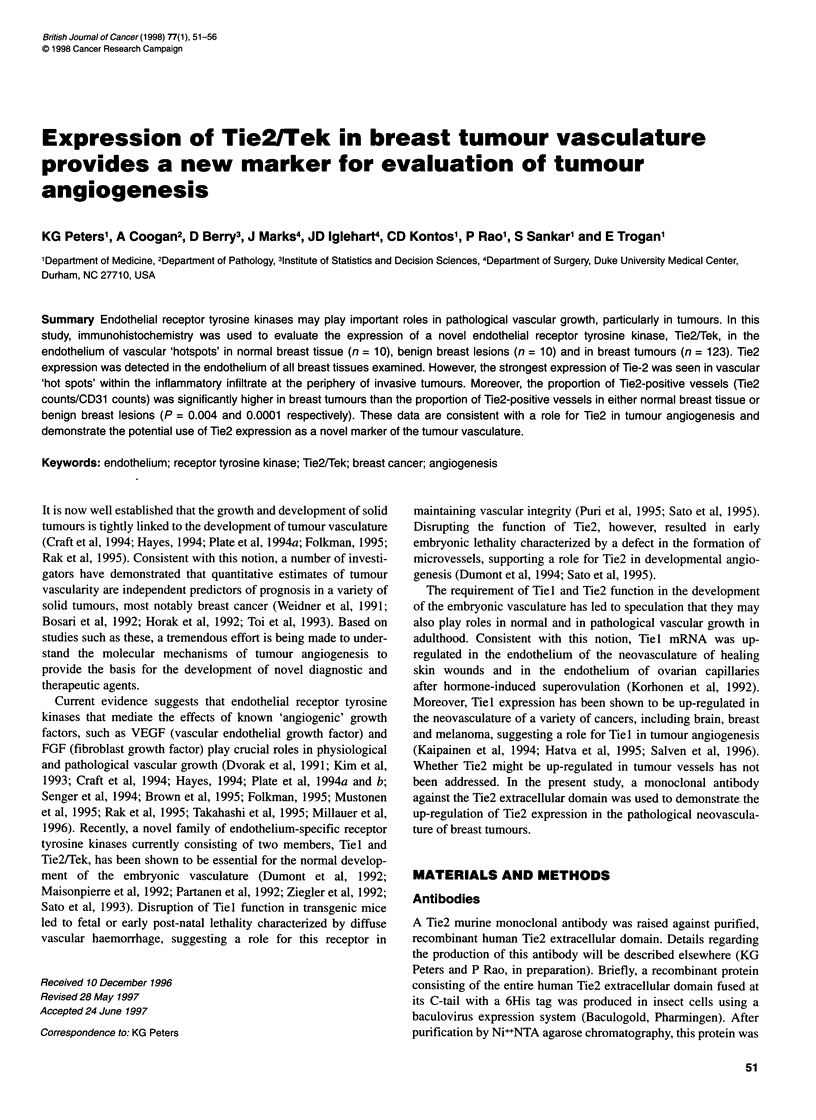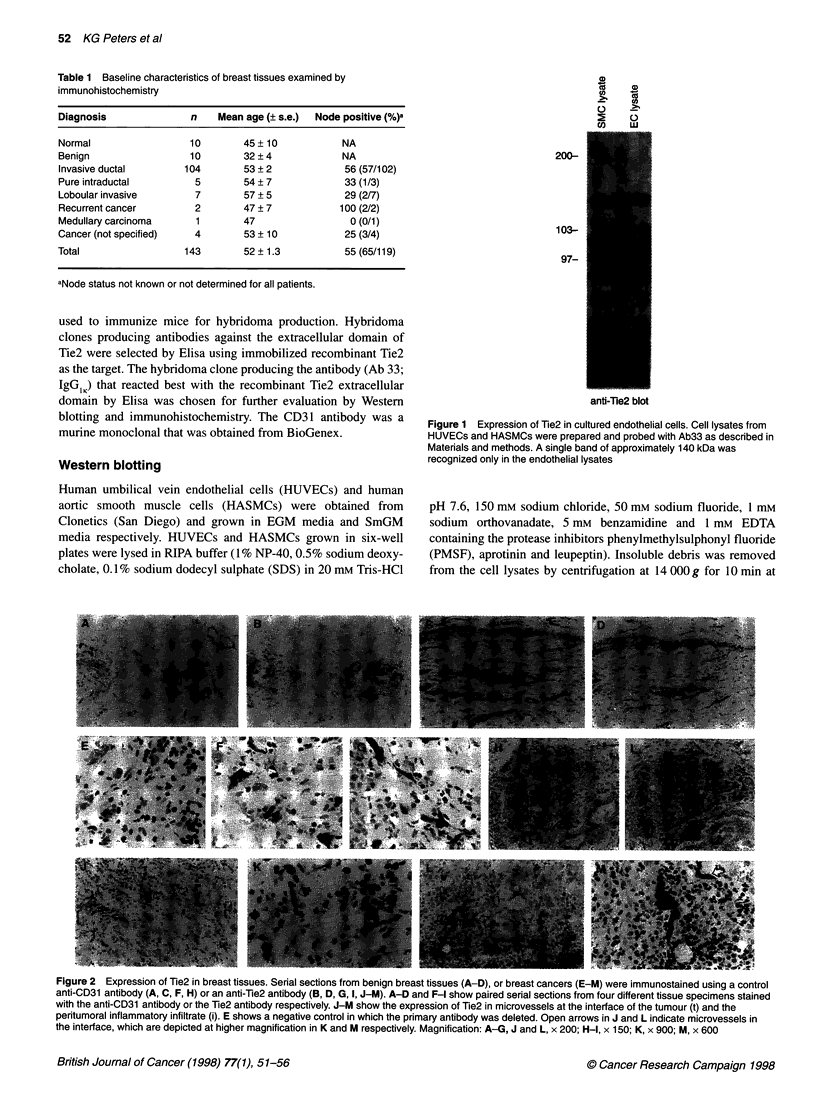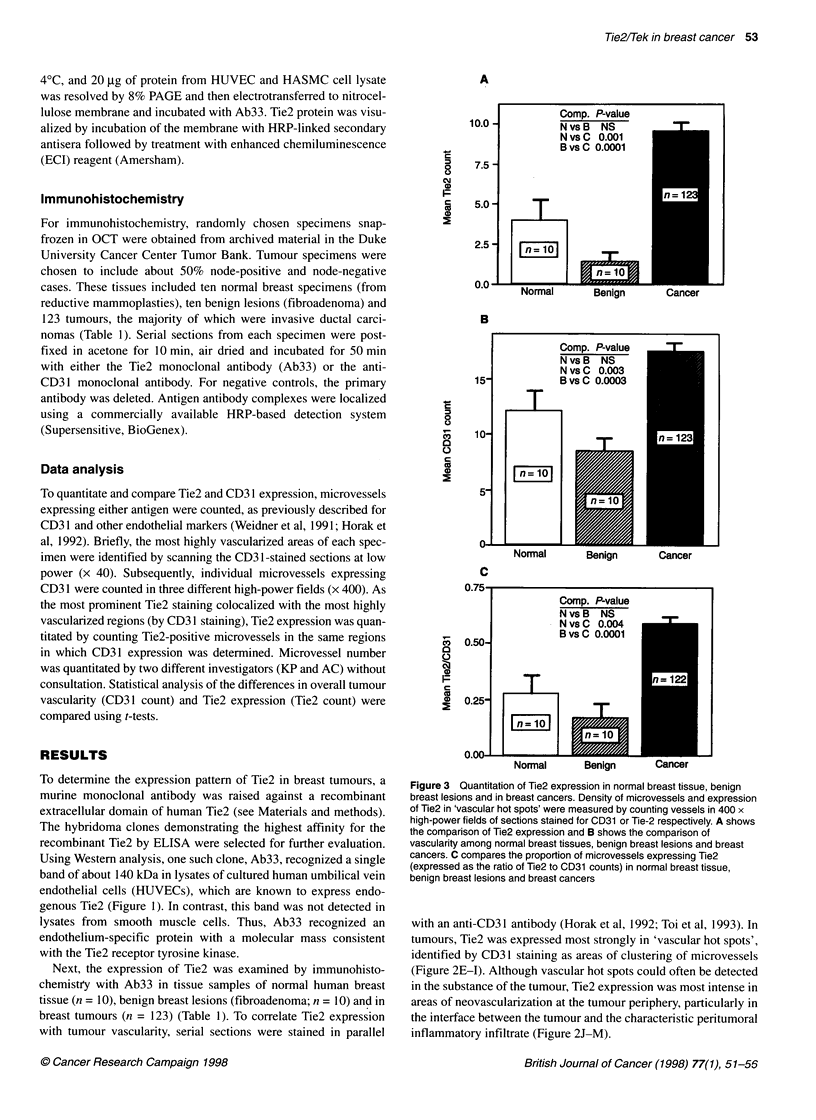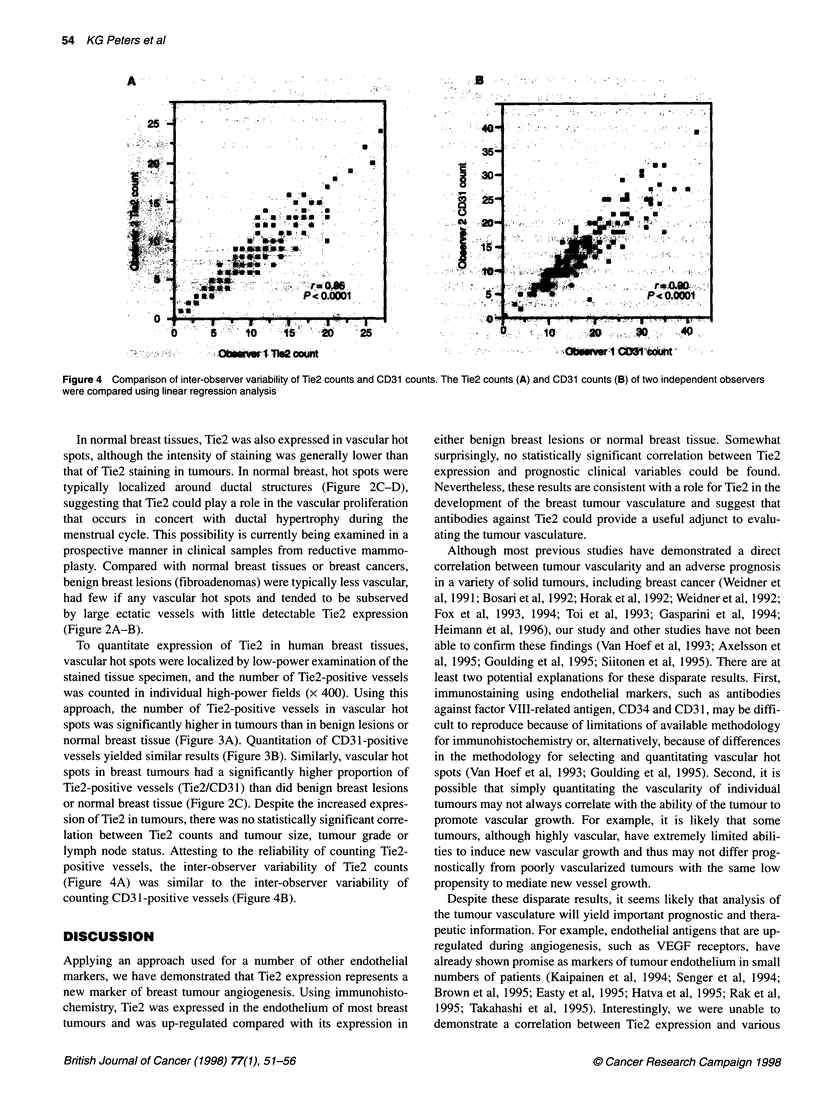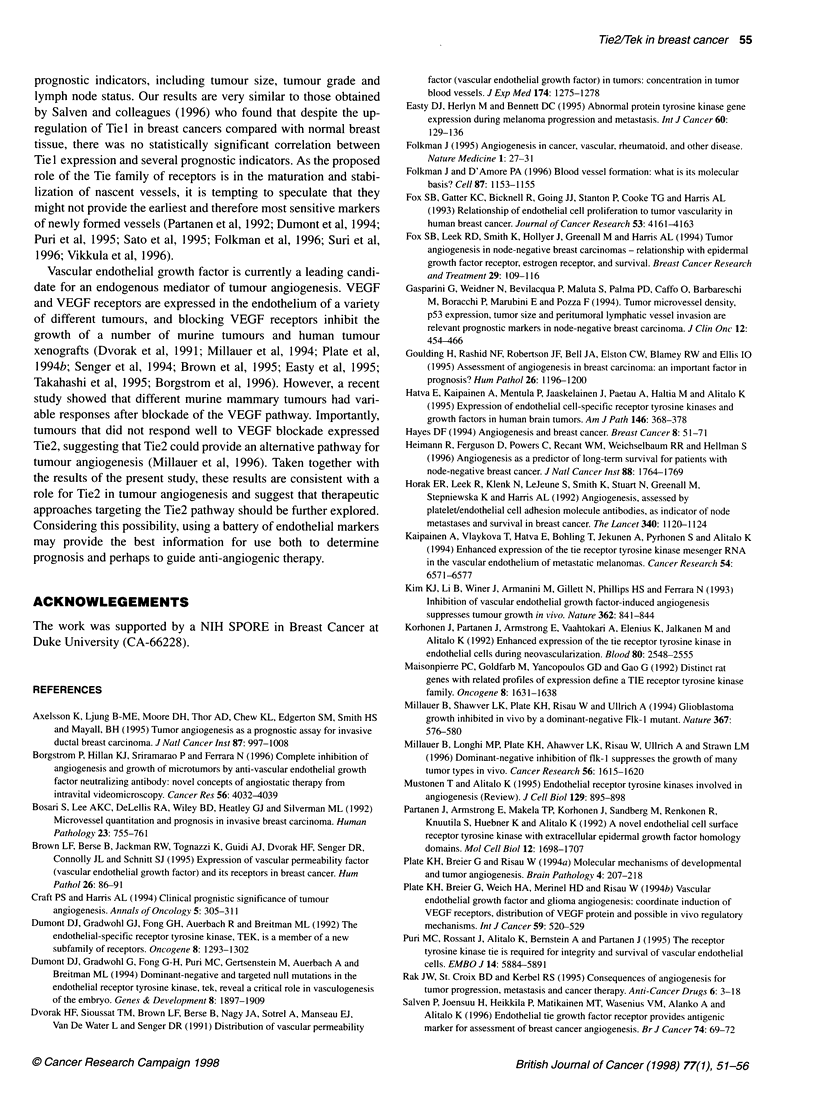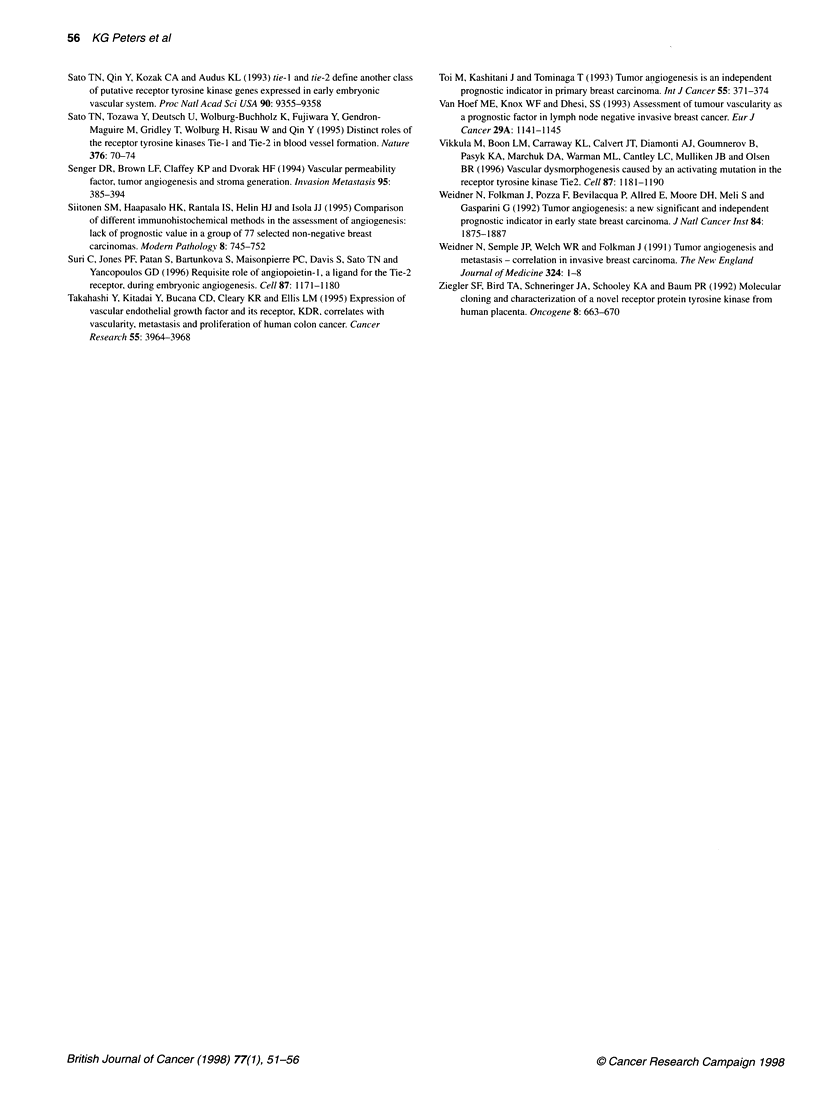# Expression of Tie2/Tek in breast tumour vasculature provides a new marker for evaluation of tumour angiogenesis.

**DOI:** 10.1038/bjc.1998.8

**Published:** 1998

**Authors:** K. G. Peters, A. Coogan, D. Berry, J. Marks, J. D. Iglehart, C. D. Kontos, P. Rao, S. Sankar, E. Trogan

**Affiliations:** Department of Medicine, Duke University Medical Center, Durham, NC 27710, USA.

## Abstract

**Images:**


British Journal of Cancer (1998) 77(1), 51–56
© 1998 Cancer Research Campaign

### Expression of Tie2/Tek in breast tumour vasculature provides a new marker for evaluation of tumour angiogenesis

**KG Peters[1], A Coogan[2], D Berry[3], J Marks[4], JD Iglehart[4], CD Kontos[1], P Rao[1], S Sankar[1] and E Trogan[1]**

[1]Department of Medicine, [2]Department of Pathology, [3]Institute of Statistics and Decision Sciences, [4]Department of Surgery, Duke University Medical Center, Durham, NC 27710, USA

**Summary** Endothelial receptor tyrosine kinases may play important roles in pathological vascular growth, particularly in tumours. In this study, immunohistochemistry was used to evaluate the expression of a novel endothelial receptor tyrosine kinase, Tie2/Tek, in the endothelium of vascular 'hotspots' in normal breast tissue ($n$ = 10), benign breast lesions ($n$ = 10) and in breast tumours ($n$ = 123). Tie2 expression was detected in the endothelium of all breast tissues examined. However, the strongest expression of Tie-2 was seen in vascular 'hot spots' within the inflammatory infiltrate at the periphery of invasive tumours. Moreover, the proportion of Tie2-positive vessels (Tie2 counts/CD31 counts) was significantly higher in breast tumours than the proportion of Tie2-positive vessels in either normal breast tissue or benign breast lesions ($P$ = 0.004 and 0.0001 respectively). These data are consistent with a role for Tie2 in tumour angiogenesis and demonstrate the potential use of Tie2 expression as a novel marker of the tumour vasculature.

**Keywords:** endothelium; receptor tyrosine kinase; Tie2/Tek; breast cancer; angiogenesis

It is now well established that the growth and development of solid tumours is tightly linked to the development of tumour vasculature (Craft et al, 1994; Hayes, 1994; Plate et al, 1994*a*; Folkman, 1995; Rak et al, 1995). Consistent with this notion, a number of investigators have demonstrated that quantitative estimates of tumour vascularity are independent predictors of prognosis in a variety of solid tumours, most notably breast cancer (Weidner et al, 1991; Bosari et al, 1992; Horak et al, 1992; Toi et al, 1993). Based on studies such as these, a tremendous effort is being made to understand the molecular mechanisms of tumour angiogenesis to provide the basis for the development of novel diagnostic and therapeutic agents.

Current evidence suggests that endothelial receptor tyrosine kinases that mediate the effects of known 'angiogenic' growth factors, such as VEGF (vascular endothelial growth factor) and FGF (fibroblast growth factor) play crucial roles in physiological and pathological vascular growth (Dvorak et al, 1991; Kim et al, 1993; Craft et al, 1994; Hayes, 1994; Plate et al, 1994*a* and *b*; Senger et al, 1994; Brown et al, 1995; Folkman, 1995; Mustonen et al, 1995; Rak et al, 1995; Takahashi et al, 1995; Millauer et al, 1996). Recently, a novel family of endothelium-specific receptor tyrosine kinases currently consisting of two members, Tie1 and Tie2/Tek, has been shown to be essential for the normal development of the embryonic vasculature (Dumont et al, 1992; Maisonpierre et al, 1992; Partanen et al, 1992; Ziegler et al, 1992; Sato et al, 1993). Disruption of Tie1 function in transgenic mice led to fetal or early post-natal lethality characterized by diffuse vascular haemorrhage, suggesting a role for this receptor in maintaining vascular integrity (Puri et al, 1995; Sato et al, 1995). Disrupting the function of Tie2, however, resulted in early embryonic lethality characterized by a defect in the formation of microvessels, supporting a role for Tie2 in developmental angiogenesis (Dumont et al, 1994; Sato et al, 1995).

The requirement of Tie1 and Tie2 function in the development of the embryonic vasculature has led to speculation that they may also play roles in normal and in pathological vascular growth in adulthood. Consistent with this notion, Tie1 mRNA was up-regulated in the endothelium of the neovasculature of healing skin wounds and in the endothelium of ovarian capillaries after hormone-induced superovulation (Korhonen et al, 1992). Moreover, Tie1 expression has been shown to be up-regulated in the neovasculature of a variety of cancers, including brain, breast and melanoma, suggesting a role for Tie1 in tumour angiogenesis (Kaipainen et al, 1994; Hatva et al, 1995; Salven et al, 1996). Whether Tie2 might be up-regulated in tumour vessels has not been addressed. In the present study, a monoclonal antibody against the Tie2 extracellular domain was used to demonstrate the up-regulation of Tie2 expression in the pathological neovasculature of breast tumours.

*Received 10 December 1996*
*Revised 28 May 1997*
*Accepted 24 June 1997*

*Correspondence to:* KG Peters

### MATERIALS AND METHODS

### Antibodies

A Tie2 murine monoclonal antibody was raised against purified, recombinant human Tie2 extracellular domain. Details regarding the production of this antibody will be described elsewhere (KG Peters and P Rao, in preparation). Briefly, a recombinant protein consisting of the entire human Tie2 extracellular domain fused at its C-tail with a 6His tag was produced in insect cells using a baculovirus expression system (Baculogold, Pharmingen). After purification by $Ni^{++}$NTA agarose chromatography, this protein was

**Table 1** Baseline characteristics of breast tissues examined by immunohistochemistry

| Diagnosis | *n* | Mean age (± s.e.) | Node positive (%)[a] |
|---|---|---|---|
| Normal | 10 | 45 ± 10 | NA |
| Benign | 10 | 32 ± 4 | NA |
| Invasive ductal | 104 | 53 ± 2 | 56 (57/102) |
| Pure intraductal | 5 | 54 ± 7 | 33 (1/3) |
| Loboular invasive | 7 | 57 ± 5 | 29 (2/7) |
| Recurrent cancer | 2 | 47 ± 7 | 100 (2/2) |
| Medullary carcinoma | 1 | 47 | 0 (0/1) |
| Cancer (not specified) | 4 | 53 ± 10 | 25 (3/4) |
| Total | 143 | 52 ± 1.3 | 55 (65/119) |

[a]Node status not known or not determined for all patients.

used to immunize mice for hybridoma production. Hybridoma clones producing antibodies against the extracellular domain of Tie2 were selected by Elisa using immobilized recombinant Tie2 as the target. The hybridoma clone producing the antibody (Ab 33; $IgG_{1\kappa}$) that reacted best with the recombinant Tie2 extracellular domain by Elisa was chosen for further evaluation by Western blotting and immunohistochemistry. The CD31 antibody was a murine monoclonal that was obtained from BioGenex.

### Western blotting

Human umbilical vein endothelial cells (HUVECs) and human aortic smooth muscle cells (HASMCs) were obtained from Clonetics (San Diego) and grown in EGM media and SmGM media respectively. HUVECs and HASMCs grown in six-well plates were lysed in RIPA buffer (1% NP-40, 0.5% sodium deoxycholate, 0.1% sodium dodecyl sulphate (SDS) in 20 mM Tris-HCl pH 7.6, 150 mM sodium chloride, 50 mM sodium fluoride, 1 mM sodium orthovanadate, 5 mM benzamidine and 1 mM EDTA containing the protease inhibitors phenylmethylsulphonyl fluoride (PMSF), aprotinin and leupeptin). Insoluble debris was removed from the cell lysates by centrifugation at 14 000 *g* for 10 min at

**Figure 1** Expression of Tie2 in cultured endothelial cells. Cell lysates from HUVECs and HASMCs were prepared and probed with Ab33 as described in Materials and methods. A single band of approximately 140 kDa was recognized only in the endothelial lysates

**Figure 2** Expression of Tie2 in breast tissues. Serial sections from benign breast tissues (**A–D**), or breast cancers (**E–M**) were immunostained using a control anti-CD31 antibody (**A**, **C**, **F**, **H**) or an anti-Tie2 antibody (**B**, **D**, **G**, **I**, **J–M**). **A–D** and **F–I** show paired serial sections from four different tissue specimens stained with the anti-CD31 antibody or the Tie2 antibody respectively. **J–M** show the expression of Tie2 in microvessels at the interface of the tumour (t) and the peritumoral inflammatory infiltrate (i). **E** shows a negative control in which the primary antibody was deleted. Open arrows in **J** and **L** indicate microvessels in the interface, which are depicted at higher magnification in **K** and **M** respectively. Magnification: **A–G**, **J** and **L**, × 200; **H–I**, × 150; **K**, × 900; **M**, × 600

© Cancer Research Campaign 1998

4°C, and 20 μg of protein from HUVEC and HASMC cell lysate was resolved by 8% PAGE and then electrotransferred to nitrocellulose membrane and incubated with Ab33. Tie2 protein was visualized by incubation of the membrane with HRP-linked secondary antisera followed by treatment with enhanced chemiluminescence (ECI) reagent (Amersham).

### Immunohistochemistry

For immunohistochemistry, randomly chosen specimens snap-frozen in OCT were obtained from archived material in the Duke University Cancer Center Tumor Bank. Tumour specimens were chosen to include about 50% node-positive and node-negative cases. These tissues included ten normal breast specimens (from reductive mammoplasties), ten benign lesions (fibroadenoma) and 123 tumours, the majority of which were invasive ductal carcinomas (Table 1). Serial sections from each specimen were post-fixed in acetone for 10 min, air dried and incubated for 50 min with either the Tie2 monoclonal antibody (Ab33) or the anti-CD31 monoclonal antibody. For negative controls, the primary antibody was deleted. Antigen antibody complexes were localized using a commercially available HRP-based detection system (Supersensitive, BioGenex).

### Data analysis

To quantitate and compare Tie2 and CD31 expression, microvessels expressing either antigen were counted, as previously described for CD31 and other endothelial markers (Weidner et al, 1991; Horak et al, 1992). Briefly, the most highly vascularized areas of each specimen were identified by scanning the CD31-stained sections at low power (× 40). Subsequently, individual microvessels expressing CD31 were counted in three different high-power fields (× 400). As the most prominent Tie2 staining colocalized with the most highly vascularized regions (by CD31 staining), Tie2 expression was quantitated by counting Tie2-positive microvessels in the same regions in which CD31 expression was determined. Microvessel number was quantitated by two different investigators (KP and AC) without consultation. Statistical analysis of the differences in overall tumour vascularity (CD31 count) and Tie2 expression (Tie2 count) were compared using *t*-tests.

### RESULTS

To determine the expression pattern of Tie2 in breast tumours, a murine monoclonal antibody was raised against a recombinant extracellular domain of human Tie2 (see Materials and methods). The hybridoma clones demonstrating the highest affinity for the recombinant Tie2 by ELISA were selected for further evaluation. Using Western analysis, one such clone, Ab33, recognized a single band of about 140 kDa in lysates of cultured human umbilical vein endothelial cells (HUVECs), which are known to express endogenous Tie2 (Figure 1). In contrast, this band was not detected in lysates from smooth muscle cells. Thus, Ab33 recognized an endothelium-specific protein with a molecular mass consistent with the Tie2 receptor tyrosine kinase.

Next, the expression of Tie2 was examined by immunohistochemistry with Ab33 in tissue samples of normal human breast tissue ($n$ = 10), benign breast lesions (fibroadenoma; $n$ = 10) and in breast tumours ($n$ = 123) (Table 1). To correlate Tie2 expression with tumour vascularity, serial sections were stained in parallel with an anti-CD31 antibody (Horak et al, 1992; Toi et al, 1993). In tumours, Tie2 was expressed most strongly in 'vascular hot spots', identified by CD31 staining as areas of clustering of microvessels (Figure 2E–I). Although vascular hot spots could often be detected in the substance of the tumour, Tie2 expression was most intense in areas of neovascularization at the tumour periphery, particularly in the interface between the tumour and the characteristic peritumoral inflammatory infiltrate (Figure 2J–M).

**Figure 3** Quantitation of Tie2 expression in normal breast tissue, benign breast lesions and in breast cancers. Density of microvessels and expression of Tie2 in 'vascular hot spots' were measured by counting vessels in 400 × high-power fields of sections stained for CD31 or Tie-2 respectively. **A** shows the comparison of Tie2 expression and **B** shows the comparison of vascularity among normal breast tissues, benign breast lesions and breast cancers. **C** compares the proportion of microvessels expressing Tie2 (expressed as the ratio of Tie2 to CD31 counts) in normal breast tissue, benign breast lesions and breast cancers

© Cancer Research Campaign 1998

**Figure 4** Comparison of inter-observer variability of Tie2 counts and CD31 counts. The Tie2 counts (**A**) and CD31 counts (**B**) of two independent observers were compared using linear regression analysis

In normal breast tissues, Tie2 was also expressed in vascular hot spots, although the intensity of staining was generally lower than that of Tie2 staining in tumours. In normal breast, hot spots were typically localized around ductal structures (Figure 2C–D), suggesting that Tie2 could play a role in the vascular proliferation that occurs in concert with ductal hypertrophy during the menstrual cycle. This possibility is currently being examined in a prospective manner in clinical samples from reductive mammoplasty. Compared with normal breast tissues or breast cancers, benign breast lesions (fibroadenomas) were typically less vascular, had few if any vascular hot spots and tended to be subserved by large ectatic vessels with little detectable Tie2 expression (Figure 2A–B).

To quantitate expression of Tie2 in human breast tissues, vascular hot spots were localized by low-power examination of the stained tissue specimen, and the number of Tie2-positive vessels was counted in individual high-power fields (× 400). Using this approach, the number of Tie2-positive vessels in vascular hot spots was significantly higher in tumours than in benign lesions or normal breast tissue (Figure 3A). Quantitation of CD31-positive vessels yielded similar results (Figure 3B). Similarly, vascular hot spots in breast tumours had a significantly higher proportion of Tie2-positive vessels (Tie2/CD31) than did benign breast lesions or normal breast tissue (Figure 2C). Despite the increased expression of Tie2 in tumours, there was no statistically significant correlation between Tie2 counts and tumour size, tumour grade or lymph node status. Attesting to the reliability of counting Tie2-positive vessels, the inter-observer variability of Tie2 counts (Figure 4A) was similar to the inter-observer variability of counting CD31-positive vessels (Figure 4B).

### DISCUSSION

Applying an approach used for a number of other endothelial markers, we have demonstrated that Tie2 expression represents a new marker of breast tumour angiogenesis. Using immunohistochemistry, Tie2 was expressed in the endothelium of most breast tumours and was up-regulated compared with its expression in either benign breast lesions or normal breast tissue. Somewhat surprisingly, no statistically significant correlation between Tie2 expression and prognostic clinical variables could be found. Nevertheless, these results are consistent with a role for Tie2 in the development of the breast tumour vasculature and suggest that antibodies against Tie2 could provide a useful adjunct to evaluating the tumour vasculature.

Although most previous studies have demonstrated a direct correlation between tumour vascularity and an adverse prognosis in a variety of solid tumours, including breast cancer (Weidner et al, 1991; Bosari et al, 1992; Horak et al, 1992; Weidner et al, 1992; Fox et al, 1993, 1994; Toi et al, 1993; Gasparini et al, 1994; Heimann et al, 1996), our study and other studies have not been able to confirm these findings (Van Hoef et al, 1993; Axelsson et al, 1995; Goulding et al, 1995; Siitonen et al, 1995). There are at least two potential explanations for these disparate results. First, immunostaining using endothelial markers, such as antibodies against factor VIII-related antigen, CD34 and CD31, may be difficult to reproduce because of limitations of available methodology for immunohistochemistry or, alternatively, because of differences in the methodology for selecting and quantitating vascular hot spots (Van Hoef et al, 1993; Goulding et al, 1995). Second, it is possible that simply quantitating the vascularity of individual tumours may not always correlate with the ability of the tumour to promote vascular growth. For example, it is likely that some tumours, although highly vascular, have extremely limited abilities to induce new vascular growth and thus may not differ prognostically from poorly vascularized tumours with the same low propensity to mediate new vessel growth.

Despite these disparate results, it seems likely that analysis of the tumour vasculature will yield important prognostic and therapeutic information. For example, endothelial antigens that are up-regulated during angiogenesis, such as VEGF receptors, have already shown promise as markers of tumour endothelium in small numbers of patients (Kaipainen et al, 1994; Senger et al, 1994; Brown et al, 1995; Easty et al, 1995; Hatva et al, 1995; Rak et al, 1995; Takahashi et al, 1995). Interestingly, we were unable to demonstrate a correlation between Tie2 expression and various

© Cancer Research Campaign 1998

prognostic indicators, including tumour size, tumour grade and lymph node status. Our results are very similar to those obtained by Salven and colleagues (1996) who found that despite the upregulation of Tie1 in breast cancers compared with normal breast tissue, there was no statistically significant correlation between Tie1 expression and several prognostic indicators. As the proposed role of the Tie family of receptors is in the maturation and stabilization of nascent vessels, it is tempting to speculate that they might not provide the earliest and therefore most sensitive markers of newly formed vessels (Partanen et al, 1992; Dumont et al, 1994; Puri et al, 1995; Sato et al, 1995; Folkman et al, 1996; Suri et al, 1996; Vikkula et al, 1996).

Vascular endothelial growth factor is currently a leading candidate for an endogenous mediator of tumour angiogenesis. VEGF and VEGF receptors are expressed in the endothelium of a variety of different tumours, and blocking VEGF receptors inhibit the growth of a number of murine tumours and human tumour xenografts (Dvorak et al, 1991; Millauer et al, 1994; Plate et al, 1994*b*; Senger et al, 1994; Brown et al, 1995; Easty et al, 1995; Takahashi et al, 1995; Borgstrom et al, 1996). However, a recent study showed that different murine mammary tumours had variable responses after blockade of the VEGF pathway. Importantly, tumours that did not respond well to VEGF blockade expressed Tie2, suggesting that Tie2 could provide an alternative pathway for tumour angiogenesis (Millauer et al, 1996). Taken together with the results of the present study, these results are consistent with a role for Tie2 in tumour angiogenesis and suggest that therapeutic approaches targeting the Tie2 pathway should be further explored. Considering this possibility, using a battery of endothelial markers may provide the best information for use both to determine prognosis and perhaps to guide anti-angiogenic therapy.

### ACKNOWLEGEMENTS

The work was supported by a NIH SPORE in Breast Cancer at Duke University (CA-66228).

### REFERENCES

Axelsson K, Ljung B-ME, Moore DH, Thor AD, Chew KL, Edgerton SM, Smith HS and Mayall, BH (1995) Tumor angiogenesis as a prognostic assay for invasive ductal breast carcinoma. *J Natl Cancer Inst* **87**: 997–1008

Borgstrom P, Hillan KJ, Sriramarao P and Ferrara N (1996) Complete inhibition of angiogenesis and growth of microtumors by anti-vascular endothelial growth factor neutralizing antibody: novel concepts of angiostatic therapy from intravital videomicroscopy. *Cancer Res* **56**: 4032–4039

Bosari S, Lee AKC, DeLellis RA, Wiley BD, Heatley GJ and Silverman ML (1992) Microvessel quantitation and prognosis in invasive breast carcinoma. *Human Pathology* **23**: 755–761

Brown LF, Berse B, Jackman RW, Tognazzi K, Guidi AJ, Dvorak HF, Senger DR, Connolly JL and Schnitt SJ (1995) Expression of vascular permeability factor (vascular endothelial growth factor) and its receptors in breast cancer. *Hum Pathol* **26**: 86–91

Craft PS and Harris AL (1994) Clinical prognistic significance of tumour angiogenesis. *Annals of Oncology* **5**: 305–311

Dumont DJ, Gradwohl GJ, Fong GH, Auerbach R and Breitman ML (1992) The endothelial-specific receptor tyrosine kinase, TEK, is a member of a new subfamily of receptors. *Oncogene* **8**: 1293–1302

Dumont DJ, Gradwohl G, Fong G-H, Puri MC, Gertsenstein M, Auerbach A and Breitman ML (1994) Dominant-negative and targeted null mutations in the endothelial receptor tyrosine kinase, tek, reveal a critical role in vasculogenesis of the embryo. *Genes & Development* **8**: 1897–1909

Dvorak HF, Sioussat TM, Brown LF, Berse B, Nagy JA, Sotrel A, Manseau EJ, Van De Water L and Senger DR (1991) Distribution of vascular permeability factor (vascular endothelial growth factor) in tumors: concentration in tumor blood vessels. *J Exp Med* **174**: 1275–1278

Easty DJ, Herlyn M and Bennett DC (1995) Abnormal protein tyrosine kinase gene expression during melanoma progression and metastasis. *Int J Cancer* **60**: 129–136

Folkman J (1995) Angiogenesis in cancer, vascular, rheumatoid, and other disease. *Nature Medicine* **1**: 27–31

Folkman J and D'Amore PA (1996) Blood vessel formation: what is its molecular basis? *Cell* **87**: 1153–1155

Fox SB, Gatter KC, Bicknell R, Going JJ, Stanton P, Cooke TG and Harris AL (1993) Relationship of endothelial cell proliferation to tumor vascularity in human breast cancer. *Journal of Cancer Research* **53**: 4161–4163

Fox SB, Leek RD, Smith K, Hollyer J, Greenall M and Harris AL (1994) Tumor angiogenesis in node-negative breast carcinomas – relationship with epidermal growth factor receptor, estrogen receptor, and survival. *Breast Cancer Research and Treatment* **29**: 109–116

Gasparini G, Weidner N, Bevilacqua P, Maluta S, Palma PD, Caffo O, Barbareschi M, Boracchi P, Marubini E and Pozza F (1994). Tumor microvessel density, p53 expression, tumor size and peritumoral lymphatic vessel invasion are relevant prognostic markers in node-negative breast carcinoma. *J Clin Onc* **12**: 454–466

Goulding H, Rashid NF, Robertson JF, Bell JA, Elston CW, Blamey RW and Ellis IO (1995) Assessment of angiogenesis in breast carcinoma: an important factor in prognosis? *Hum Pathol* **26**: 1196–1200

Hatva E, Kaipainen A, Mentula P, Jaaskelainen J, Paetau A, Haltia M and Alitalo K (1995) Expression of endothelial cell-specific receptor tyrosine kinases and growth factors in human brain tumors. *Am J Path* **146**: 368–378

Hayes DF (1994) Angiogenesis and breast cancer. *Breast Cancer* **8**: 51–71

Heimann R, Ferguson D, Powers C, Recant WM, Weichselbaum RR and Hellman S (1996) Angiogenesis as a predictor of long-term survival for patients with node-negative breast cancer. *J Natl Cancer Inst* **88**: 1764–1769

Horak ER, Leek R, Klenk N, LeJeune S, Smith K, Stuart N, Greenall M, Stepniewska K and Harris AL (1992) Angiogenesis, assessed by platelet/endothelial cell adhesion molecule antibodies, as indicator of node metastases and survival in breast cancer. *The Lancet* **340**: 1120–1124

Kaipainen A, Vlaykova T, Hatva E, Bohling T, Jekunen A, Pyrhonen S and Alitalo K (1994) Enhanced expression of the tie receptor tyrosine kinase mesenger RNA in the vascular endothelium of metastatic melanomas. *Cancer Research* **54**: 6571–6577

Kim KJ, Li B, Winer J, Armanini M, Gillett N, Phillips HS and Ferrara N (1993) Inhibition of vascular endothelial growth factor-induced angiogenesis suppresses tumour growth *in vivo*. *Nature* **362**: 841–844

Korhonen J, Partanen J, Armstrong E, Vaahtokari A, Elenius K, Jalkanen M and Alitalo K (1992) Enhanced expression of the tie receptor tyrosine kinase in endothelial cells during neovascularization. *Blood* **80**: 2548–2555

Maisonpierre PC, Goldfarb M, Yancopoulos GD and Gao G (1992) Distinct rat genes with related profiles of expression define a TIE receptor tyrosine kinase family. *Oncogene* **8**: 1631–1638

Millauer B, Shawver LK, Plate KH, Risau W and Ullrich A (1994) Glioblastoma growth inhibited in vivo by a dominant-negative Flk-1 mutant. *Nature* **367**: 576–580

Millauer B, Longhi MP, Plate KH, Ahawver LK, Risau W, Ullrich A and Strawn LM (1996) Dominant-negative inhibition of flk-1 suppresses the growth of many tumor types in vivo. *Cancer Research* **56**: 1615–1620

Mustonen T and Alitalo K (1995) Endothelial receptor tyrosine kinases involved in angiogenesis (Review). *J Cell Biol* **129**: 895–898

Partanen J, Armstrong E, Makela TP, Korhonen J, Sandberg M, Renkonen R, Knuutila S, Huebner K and Alitalo K (1992) A novel endothelial cell surface receptor tyrosine kinase with extracellular epidermal growth factor homology domains. *Mol Cell Biol* **12**: 1698–1707

Plate KH, Breier G and Risau W (1994*a*) Molecular mechanisms of developmental and tumor angiogenesis. *Brain Pathology* **4**: 207–218

Plate KH, Breier G, Weich HA, Merinel HD and Risau W (1994*b*) Vascular endothelial growth factor and glioma angiogenesis: coordinate induction of VEGF receptors, distribution of VEGF protein and possible in vivo regulatory mechanisms. *Int J Cancer* **59**: 520–529

Puri MC, Rossant J, Alitalo K, Bernstein A and Partanen J (1995) The receptor tyrosine kinase tie is required for integrity and survival of vascular endothelial cells. *EMBO J* **14**: 5884–5891

Rak JW, St. Croix BD and Kerbel RS (1995) Consequences of angiogenesis for tumor progression, metastasis and cancer therapy. *Anti-Cancer Drugs* **6**: 3–18

Salven P, Joensuu H, Heikkila P, Matikainen MT, Wasenius VM, Alanko A and Alitalo K (1996) Endothelial tie growth factor receptor provides antigenic marker for assessment of breast cancer angiogenesis. *Br J Cancer* **74**: 69–72

© Cancer Research Campaign 1998

Sato TN, Qin Y, Kozak CA and Audus KL (1993) *tie*-1 and *tie*-2 define another class of putative receptor tyrosine kinase genes expressed in early embryonic vascular system. *Proc Natl Acad Sci USA* **90**: 9355–9358

Sato TN, Tozawa Y, Deutsch U, Wolburg-Buchholz K, Fujiwara Y, Gendron-Maguire M, Gridley T, Wolburg H, Risau W and Qin Y (1995) Distinct roles of the receptor tyrosine kinases Tie-1 and Tie-2 in blood vessel formation. *Nature* **376**: 70–74

Senger DR, Brown LF, Claffey KP and Dvorak HF (1994) Vascular permeability factor, tumor angiogenesis and stroma generation. *Invasion Metastasis* **95**: 385–394

Siitonen SM, Haapasalo HK, Rantala IS, Helin HJ and Isola JJ (1995) Comparison of different immunohistochemical methods in the assessment of angiogenesis: lack of prognostic value in a group of 77 selected non-negative breast carcinomas. *Modern Pathology* **8**: 745–752

Suri C, Jones PF, Patan S, Bartunkova S, Maisonpierre PC, Davis S, Sato TN and Yancopoulos GD (1996) Requisite role of angiopoietin-1, a ligand for the Tie-2 receptor, during embryonic angiogenesis. *Cell* **87**: 1171–1180

Takahashi Y, Kitadai Y, Bucana CD, Cleary KR and Ellis LM (1995) Expression of vascular endothelial growth factor and its receptor, KDR, correlates with vascularity, metastasis and proliferation of human colon cancer. *Cancer Research* **55**: 3964–3968

Toi M, Kashitani J and Tominaga T (1993) Tumor angiogenesis is an independent prognostic indicator in primary breast carcinoma. *Int J Cancer* **55**: 371–374

Van Hoef ME, Knox WF and Dhesi, SS (1993) Assessment of tumour vascularity as a prognostic factor in lymph node negative invasive breast cancer. *Eur J Cancer* **29A**: 1141–1145

Vikkula M, Boon LM, Carraway KL, Calvert JT, Diamonti AJ, Goumnerov B, Pasyk KA, Marchuk DA, Warman ML, Cantley LC, Mulliken JB and Olsen BR (1996) Vascular dysmorphogenesis caused by an activating mutation in the receptor tyrosine kinase Tie2. *Cell* **87**: 1181–1190

Weidner N, Folkman J, Pozza F, Bevilacqua P, Allred E, Moore DH, Meli S and Gasparini G (1992) Tumor angiogenesis: a new significant and independent prognostic indicator in early state breast carcinoma. *J Natl Cancer Inst* **84**: 1875–1887

Weidner N, Semple JP, Welch WR and Folkman J (1991) Tumor angiogenesis and metastasis – correlation in invasive breast carcinoma. *The New England Journal of Medicine* **324**: 1–8

Ziegler SF, Bird TA, Schneringer JA, Schooley KA and Baum PR (1992) Molecular cloning and characterization of a novel receptor protein tyrosine kinase from human placenta. *Oncogene* **8**: 663–670

© Cancer Research Campaign 1998